# Dose-response relationship of MSCs as living Bio-drugs in HFrEF patients: a systematic review and meta-analysis of RCTs

**DOI:** 10.1186/s13287-024-03713-4

**Published:** 2024-06-13

**Authors:** Ziyad T. Ahmed, Maha Saad Zain Al-Abeden, Mohamed Ghaith Al Abdin, Mohamad Ayham Muqresh, Ghazi I. Al Jowf, Lars M. T. Eijssen, Khawaja Husnain Haider

**Affiliations:** 1 College of Medicine, Sulaiman Al Rajhi University, Al-Bukairiyah, 52726 Saudi Arabia; 2 Department of Public Health, College of Applied Medical Sciences, King Faisal University, Al-Ahsa, 31982 Saudi Arabia; 3 Department of Psychiatry and Neuropsychology, School for Mental Health and Neuroscience (MHeNs), Faculty of Health, Medicine and Life Sciences, Maastricht University Medical Centre, Maastricht, 6200 MD The Netherlands; 4 Department of Bioinformatics– BiGCaT, School of Nutrition and Translational Research in Metabolism (NUTRIM), Faculty of Health, Medicine and Life Sciences, Maastricht University, Maastricht, 6200 MD The Netherlands; 5 European Graduate School of Neuroscience, Maastricht University, Maastricht, 6200 MD The Netherlands

**Keywords:** Dose-response, Efficacy, Heart failure, Heart disease, Mesenchymal stem cells, Mesenchymal precursor cells, Safety

## Abstract

**Background:**

Mesenchymal stem cells (MSCs) have emerged as living biodrugs for myocardial repair and regeneration. Recent randomized controlled trials (RCTs) have reported that MSC-based therapy is safe and effective in heart failure patients; however, its dose-response relationship has yet to be established. We aimed to determine the optimal MSC dose for treating HF patients with reduced ejection fraction (EF) (HFrEF).

**Methods:**

The preferred reporting items for systematic reviews and meta-analyses (PRISMA) and Cochrane Handbook guidelines were followed. Four databases and registries, i.e., PubMed, EBSCO, clinicaltrials.gov, ICTRP, and other websites, were searched for RCTs. Eleven RCTs with 1098 participants (treatment group, *n* = 606; control group, *n* = 492) were selected based on our inclusion/exclusion criteria. Two independent assessors extracted the data and performed quality assessments. The data from all eligible studies were plotted for death, major adverse cardiac events (MACE), left ventricular ejection fraction (LVEF), left ventricular end-systolic volume (LVESV), and 6-minute walk distance (6-MWD) as safety, efficacy, and performance parameters. For dose-escalation assessment, studies were categorized as low-dose (< 100 million cells) or high-dose (≥ 100 million cells).

**Results:**

MSC-based treatment is safe across low and high doses, with nonsignificant effects. However, low-dose treatment had a more significant protective effect than high-dose treatment. Subgroup analysis revealed the superiority of low-dose treatment in improving LVEF by 3.01% (95% CI; 0.65–5.38%) compared with high-dose treatment (-0.48%; 95% CI; -2.14-1.18). MSC treatment significantly improved the 6-MWD by 26.74 m (95% CI; 3.74–49.74 m) in the low-dose treatment group and by 36.73 m (95% CI; 6.74–66.72 m) in the high-dose treatment group. The exclusion of studies using ADRCs resulted in better safety and a significant improvement in LVEF from low- and high-dose MSC treatment.

**Conclusion:**

Low-dose MSC treatment was safe and superior to high-dose treatment in restoring efficacy and functional outcomes in heart failure patients, and further analysis in a larger patient group is warranted.

**Supplementary Information:**

The online version contains supplementary material available at 10.1186/s13287-024-03713-4.

## Introduction

The prevalence of heart failure (HF) among adults in developed countries is approximately 1–2%, which significantly increases morbidity and mortality in addition to imposing a significant financial burden on the healthcare system [1]. The gravity of the problem may further be gauged from the figures, which show that the lifetime risk of HF has increased to 24%; every one in four persons is at risk of developing HF in their lifetime. Although different emerging therapies, including pharmacological and surgical options, have enhanced the survival rate, patients with HF still have significant mortality and hospitalization rates [2].

HF is characterized by the loss of a critical number of functioning cardiomyocytes (CMs), leading to left ventricular pump function deterioration. Given the inept intrinsic repair process, in addition to the limited potential of CMs to re-enter the cell cycle, dead CMs are replaced with noncontractile scar tissue, which throws the heart into a vicious remodeling cycle [4, 5]. Therefore, developing a therapeutic strategy for myocardial repair and regeneration remains one of the most critical research areas for HF therapy from a clinical perspective. Cell-based therapy, mainly using mesenchymal stem cells (MSCs), has shown promise in clinical trials and has moved to advanced assessment phases [6].

Mesodermal in origin, MSCs can be obtained from diverse tissue sources [7]. They are easy to isolate and propagate in vitro and exhibit superior cell biology, with minimal immunogenicity and excellent immunomodulatory properties [8]. In response to well-defined cues, these cells can differentiate into several types. One of the critical features of MSCs is their paracrine activity, releasing both soluble and insoluble factors in their microenvironment and promoting angiogenic and tissue repair by involving the intrinsic pool of stem/progenitor cells [9, 10]. Given their robust nature, they are physically or genetically manipulated to accentuate their repairability [11, 12]. During cardiovascular applications, they have been shown to protect the myocardium via multifactorial mechanisms involving anti-inflammatory activity, cardiomyogenic differentiation in and around infarct regions, angiogenesis, cytoprotection, and antifibrotic activity [13].

To date, no agreement exists regarding the relationship between the number of injected cells and the response regarding safety and efficacy. As the domain of cell-based therapy for cardiac regeneration continues to broaden and with the emergence of MSCs as candidates for living biodrugs, there is an urgent need to establish a dose-response relationship to determine an optimal dose for the required prognosis. The last two decades of clinical research have focused primarily on selecting the most suitable cell type [14–17]. Only a few dose-escalation studies have been reported to establish a dose-response relationship, but there are significant discrepancies in their design and findings [17–19]. While some research suggests that a lower cell dose is more effective, others have reported a direct or nonlinear relationship [20]. A recent meta-analysis of preclinical studies using MSC therapy for acute kidney injury reported no significant difference between cell doses [21]. Similarly, a meta-analysis of adipose-derived MSCs for the treatment of knee osteoarthritis reported no differences in safety or efficacy across high, moderate, and low doses. However, the rate of adverse events increases with increasing dose [22]. Subanalyses of clinical trials in which different cell doses were tested failed to predict or determine a relationship between dosage and response [23, 24]. These discrepancies and conflicting findings lead to the conclusion that the dose-response relationship might vary according to cell type, route of administration, disease, and source, among other factors, which poses a significant challenge.

Our meta-analysis was restricted to MSC-based phase II/III randomized controlled trials (RCTs) involving HF patients with reduced ejection fraction (HFrEF) only to avoid heterogeneity in the reported data between phase-I and RCTs, aiming to establish a dose-response relationship. We hypothesize that increasing the cell dose is safe and improves functional outcomes in HF patients. We followed the PRISMA guidelines for systematic reviews and meta-analyses to explore safety, efficacy, and functional clinical outcomes (i.e., death and MACEs, LVEF, LVESV, and the 6-MWD.

## Methodology

### Protocol and registration

This systemic review and meta-analysis follow the guidelines of the Preferred Reporting Items for Systematic Review and Meta-analysis (PRISMA) [25] and carries the PROSPERO identification number CRD42024501959 (registered before conducting formal research or analysis).

### Literature search and study selection process

A systematic literature search of PubMed, EBSCO, ICTRP, and clinicaltrials.gov was conducted between October and December 2023. The search strategy used Medical Subject Headings (MeSH) or text search fields. In EBSCO, ICTRP, and clinicaltrials.gov, we used the search terms “heart failure,” “congestive heart failure,” “mesenchymal stem cells,” and “mesenchymal precursor cells” in the text words with appropriate use of the Boolean operator. The search terms for PubMed included “heart failure OR congestive heart disease” AND “bone marrow mesenchymal stem cells OR bone marrow mesenchymal precursor cells.” All the references of eligible studies were screened and reviewed carefully for any potential RCTs.

### Inclusion and exclusion criteria

The inclusion criteria for this study were [1] a phase II/III randomized clinical trial [2], treatment using MSCs, and [3] HF patients only. The exclusion criteria were trials without a clear statement about the number of cells administered to the intervention group, treatment with other forms of intervention (e.g., CABG, different forms of stem cells, left ventricular assist device), preserved LVEF (pLVEF), and phase I trials irrespective of the cell type used.

### Data extraction and outcomes of interest

Two authors checked the eligibility of the studies and extracted the data into standardized Excel spreadsheets containing several relevant variables. The primary variables included intervention, cell dose, cell sampling site, country of trial origin, etiology, sample size, sex, age, cell delivery route, imaging modality, and follow-up period. In addition, the baseline, follow-up, and mean difference, along with the standard deviation, were recorded at the last follow-up for the LVEF, LVESV, and 6MWD. The number of deaths and MACEs were recorded during the last follow-up. The study’s corresponding author was approached for any missing data. However, if the corresponding author did not respond, WebPlotDigitizer was used to extract the missing values [26]. Subsequently, studies and controlled study arms with a mean dose of  <100 million and ≥ 100 million cells were assigned to the low-dose and high-dose subgroups, respectively.

### Quality assessment

The quality of the included RCTs was evaluated using the Jadad scale in three domains [27]. First, the study was awarded one point for randomization, and an additional point was awarded if the trial mentioned an appropriate randomization method. However, one point was deducted from the evaluation if the randomization was inappropriate. The second domain assessed was blinding, for which the trial was awarded one point for being double-blinded, and an additional point was added when the trial mentioned an appropriate method of double-blinding. However, one point was deducted if the blinding process was inappropriate. Finally, the third domain was the description of withdrawal or dropout during the clinical trial process, for which the trial was awarded a point dropout. Upon completion of the evaluation process, the scores ranging from zero to five were added to determine the quality score for each trial. A trial scoring 0–2 was considered of low quality, while those scoring three or more were deemed superior.

### Data synthesis and statistical analysis

Our comprehensive meta-analysis delved into clinical trials examining the dose-response effects of MSCs in treating HF patients. The study employed the LVEF, LVESV, and 6-MWD to assess the clinical effectiveness of treatment. For safety assessment, the rate of death and MACE during clinical trials were used to provide critical insights into the safety profile of the investigated treatment.

As these parameters were measured with consistent units, a weighted mean difference (WMD) meta-analysis was conducted to assess baseline to follow-up changes in LVEF, LVESV, and the 6MWD. At the same time, the risk ratio (RR) was used to compare the safety of the treatments to that of the controls. A subgroup analysis assessed the dose-response relationship and determined dose-related efficacy and safety. The significance of the results was determined using the 95% confidence interval (CI), with studies whose CI crossed the null effect line (i.e., zero) considered nonsignificant. The I-square value was used to determine between-study heterogeneity. An I-square value of < 25% indicates low heterogeneity, between 25% and 75% indicates moderate heterogeneity, while values > 75% indicate high heterogeneity. Funnel plots and Egger’s regression were used to assess the risk of publication bias. Funnel plots were visually evaluated for asymmetry around the effect line, while Egger’s regression indicated a risk of bias with p values < 0.05 and are provided in the supplementary material (Appendix 1). The study by Perin et al. was a dose-escalation trial and included three arms with different doses that were all controlled. Two of the three arms were independently assigned to the low-dose subgroup, while one was assigned to the high-dose subgroup.

Based on the quality of the included studies, a sensitivity analysis excluding low-quality studies was conducted to reassess overall and subgroup effects, as low-quality studies tend to overestimate the effect size. Additionally, studies using adipose-derived regenerative cells (ADRCs) have consistently reported no benefit. Accordingly, a sensitivity analysis of LVEF and LVESV, excluding studies using ADRCs, was conducted to assess overall and subgroup effects. An additional subgroup analysis comparing studies using ADRCs and BM-MSCs was completed, comparing the impact of cell sources in both low-dose and high-dose subgroups. The analysis was performed using the SPSS version 28 statistical package (SPSS Inc., Chicago, IL, USA).

## Results

### Literature review results

The four databases yielded 593 results. Most records were retrieved from EMBASE (*n* = 335), followed by PubMed (*n* = 197), ClinicalTrials.gov (*n* = 96), and the ICTRP (*n* = 59). The filters excluded 140 results; from the remaining records, the authors identified duplicates (*n* = 132) before screening.

**Fig. 1:**
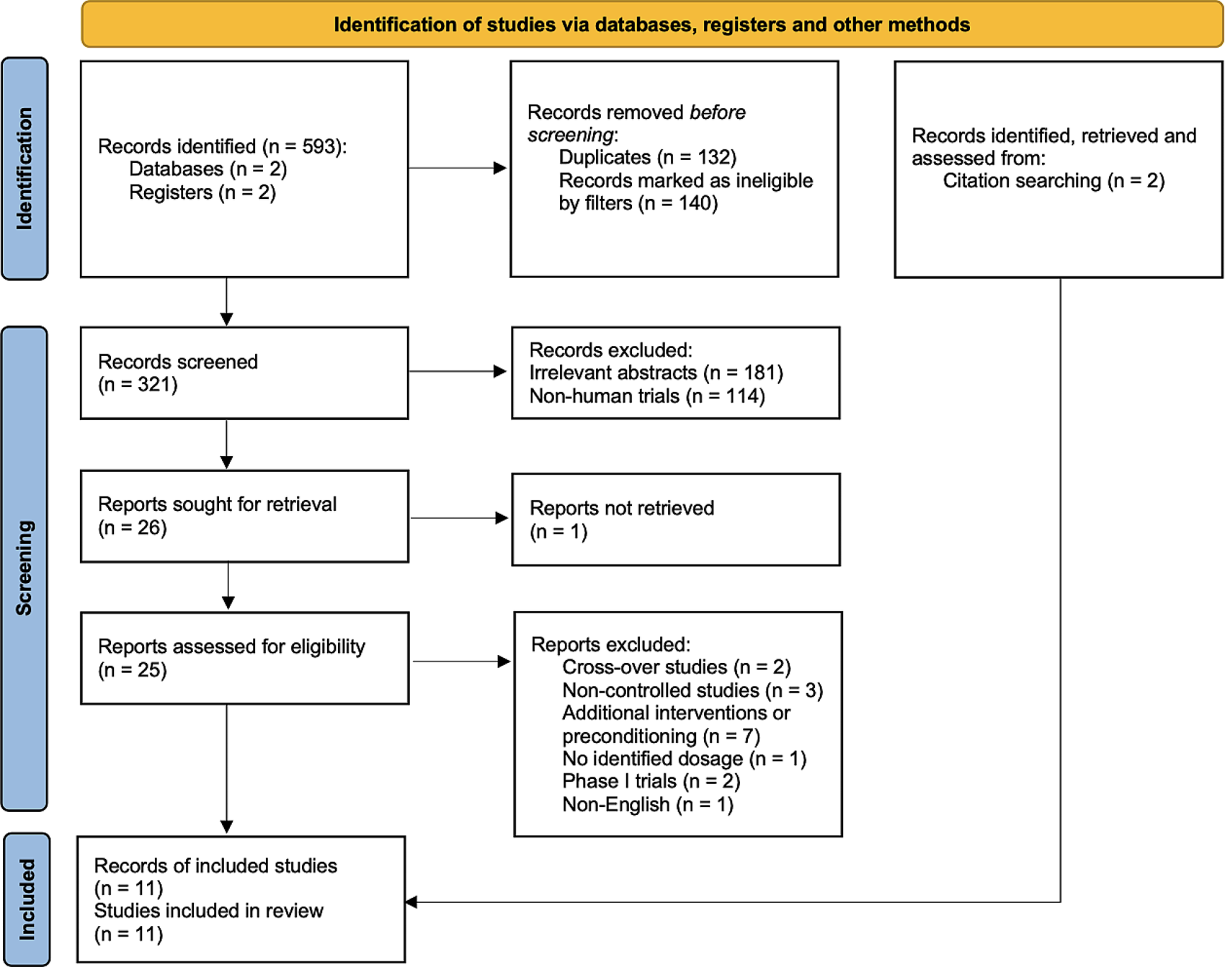
The PRISMA flow diagram for the screening and selection of eligible trials

From the screened records (*n* = 321), trials with irrelevant abstracts (*n* = 181) and nonhuman trials (*n* = 114) were excluded. A total of 26 potential candidate trials were retrieved, excluding one. The 25 retrieved trials were then screened with our inclusion/exclusion criteria, from which crossover trials (*n* = 2), noncontrolled trials (*n* = 3), trials with additional intervention (*n* = 7), trials with no identified dosage (*n* = 1), phase I trials (*n* = 2), and non-English records (*n* = 1) were excluded. Two additional trials were identified through reference searching of eligible records. Subsequently, a final total of 11 trials were included in our review for analysis (Fig. 1).

### Description of the trials included in the review and meta-analysis

Table 1 summarizes the salient features of the eleven trials in the systematic review and meta-analysis. The ATHENA trial consists of two parallel prospective trials. Of the eleven selected trials, five used a low dose, while the remaining six used a high dose of MSCs per our defined criterion of dose classification. Concerning autologous and allogenic cell sources, the trials used MSCs from bone marrow (*n* = 7), umbilical cord (*n* = 1), and adipose tissue (*n* = 3). All the trials were placebo-controlled or sham-controlled, except for Perin et al. 2015, which used a mock injection technique [28]. The total number of patients in the 11 included RCTs was 1098, with 606 patients in the intervention group and 492 patients in the control group, for an approximate ratio of 1.2:1. Male participants dominated the sample (*n* = 869). A wide range of sample sizes was noticeable between the trials, ranging from 12 to 265 in the treatment group and 12 to 272 in the control group. Most trials reported mean doses ranging from 25 million to 80 million cells in the low-dose group and from 100 million to 200 million cells in the high-dose group. Two RCTs reported the cell dose as cells/kg (1 million/kg and 1.5 million/kg). The follow-up period varied between the trials, with some reaching four years for safety assessment; however, 3 to 12 months were assessed to reduce heterogeneity. Most trials assessed LVEF using echocardiography (*n* = 7), while others used cardiac CT or MRI (*n* = 4). Transendocardial (*n* = 4) and intramyocardial (*n* = 4) routes were the standard modes of cell delivery, followed by intravenous (*n* = 2) and intracoronary (*n* = 1) routes.

**Table 1:** Description and characteristics of the included RCTs

Study	Arm	Dose (cells in millions)	Sampling site	Country	Etiology	Sample size	Males	Age	RoA	Imaging modality	F/U (mo)
**MSC-HFT** [29]	Intervention	77.5	Bone marrow (Iliac crest)	Denmark	Ischemic HF	40	36	66.1 (7.7)	IM	MRI or CT	6
	Control					20	14	64.2 (10.6)			
**Butler et al.** [30]	Intervention	1.5/kg	Bone marrow	USA	Nonischemic Cardiomyopathy	12	13	47.3 (12.8)	IV	MRI	3
	Control					12					
**Perin et al.** [28]	Intervention	25/75/150	Bone marrow (Posterior iliac crest)	USA	Ischemic or Nonischemic HF	45	2	60.1 (8.8)	TE	Echo	12
	Control					15	4	62.7 (11.2)			
**Athena Trials** [31]	Intervention	40 and 80	Abdominal adipose tissue	USA	Ischemic Cardiomyopathy	17	16	64.1 (8.2)	IM	Echo	6
	Control					14	13	65.7 (7.3)			
**RIMECARD Trial** [32]	Intervention	1/kg	Umbilical cord	Chile	Ischemic or Nonischemic Cardiomyopathy	15	12	57.33 (10.05)	IV	Echo	12
	Control					15	14	57.20 (11.64)			
**CONCERT-HF** [33]	Intervention	150	Bone marrow	USA	Ischemic Cardiomyopathy	29	27	61.7 (6.7)	TE	MRI	12
	Control					32	31	63.1 (8.8)			
**Danish phase II** [34]	Intervention	100	Abdominal adipose tissue	Denmark	Ischemic HF	54	44	67.0 (9.0)	IM	Echo	6/12
	Control					27	24	66.6 (8.1)			
**Xiao et al.** [35]	Intervention	490	Bone marrow (Posterior iliac spines)	China	Dilated Cardiomyopathy	17	12	51.6 (12.2)	IC	Echo	12
	Control					20	14	54.4 (11.6)			
**TAC-HFT** [36]	Intervention	100 or 200	Bone marrow (Iliac crest)	USA	Ischemic Cardiomyopathy	22	18	57.1 (10.6)	TE	MRI or CT	12
	Control					22	10	60 (12.0)			
**SCIENCE** [37]	Intervention	110	Abdominal adipose tissue	Denmark	Ischemic HF	90	84	66.4 (8.1)	IM	Echo	6/12
	Control					43	38	64.0 (8.8)			
**DREAM-HF** [38]	Intervention	150	Bone marrow	Canada, USA	Ischemic or Nonischemic HF	265	222	62.7 (10.9)	TE	Echo	12
	Control					272	221	62.6 (10.4)			

**Abbreviations**: Echo: Echocardiography; F/U: Follow-up; HF: Heart failure; IC: Intracoronary injection; IM: Intramyocardial injection; IV: Intravenous injection; Mo: Months; TE: RoA: Route of administration; Transendocardial injection

### Quality Assessment (risk of Bias) using the Jadad score

The Jadad score for the trials ranged between 2 and 5. Interestingly, all trials were of high quality (i.e., ≥ 3), except for the study by Xiao et al., which scored 2. Accordingly, the study was excluded from the primary meta-analysis to avoid effect overestimation. A detailed assessment of the included trials is shown in Table 2.

**Table 2:** The Jadad score for risk of bias (quality) assessment

Jadad Scale for Risk of Bias Assessment							
	Jadad Scale Items						
Study	J1	J2	J3	J4	J5	Total	Quality
**MSC-HFT**	1	1	1	1	0	4	High
**Butler et al.**	1	1	0	0	1	3	High
**Perin et al.**	1	1	1	1	1	5	High
**Athena**	1	0	1	1	1	4	High
**RIMECARD**	1	1	1	1	0	4	High
**CONCERT-HF**	1	1	1	1	1	5	High
**Danish Phase II**	1	1	1	1	0	4	High
**Xiao et al.**	1	1	0	0	0	2	Low
**TAC-HFT**	1	1	1	1	0	4	High
**SCIENCE**	1	1	1	1	1	5	High
**DREAM-HF**	1	0	1	1	1	4	High

### Meta-analysis for Safety and Efficacy parameters

#### Death and major adverse cardiac events

**Fig. 2:**
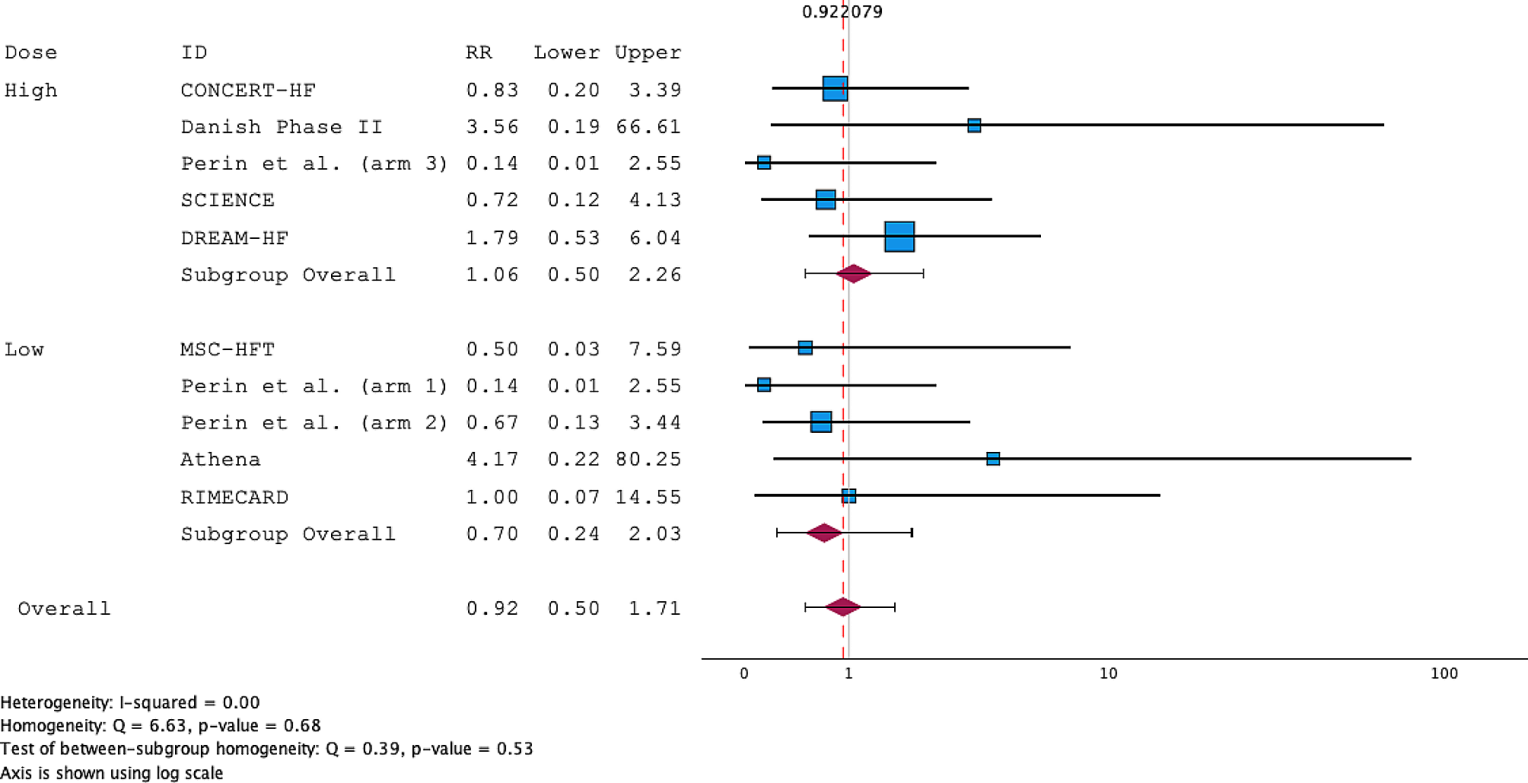
Forest plot of the risk ratio (RR) for death in the meta-analysis

**Fig. 3:**
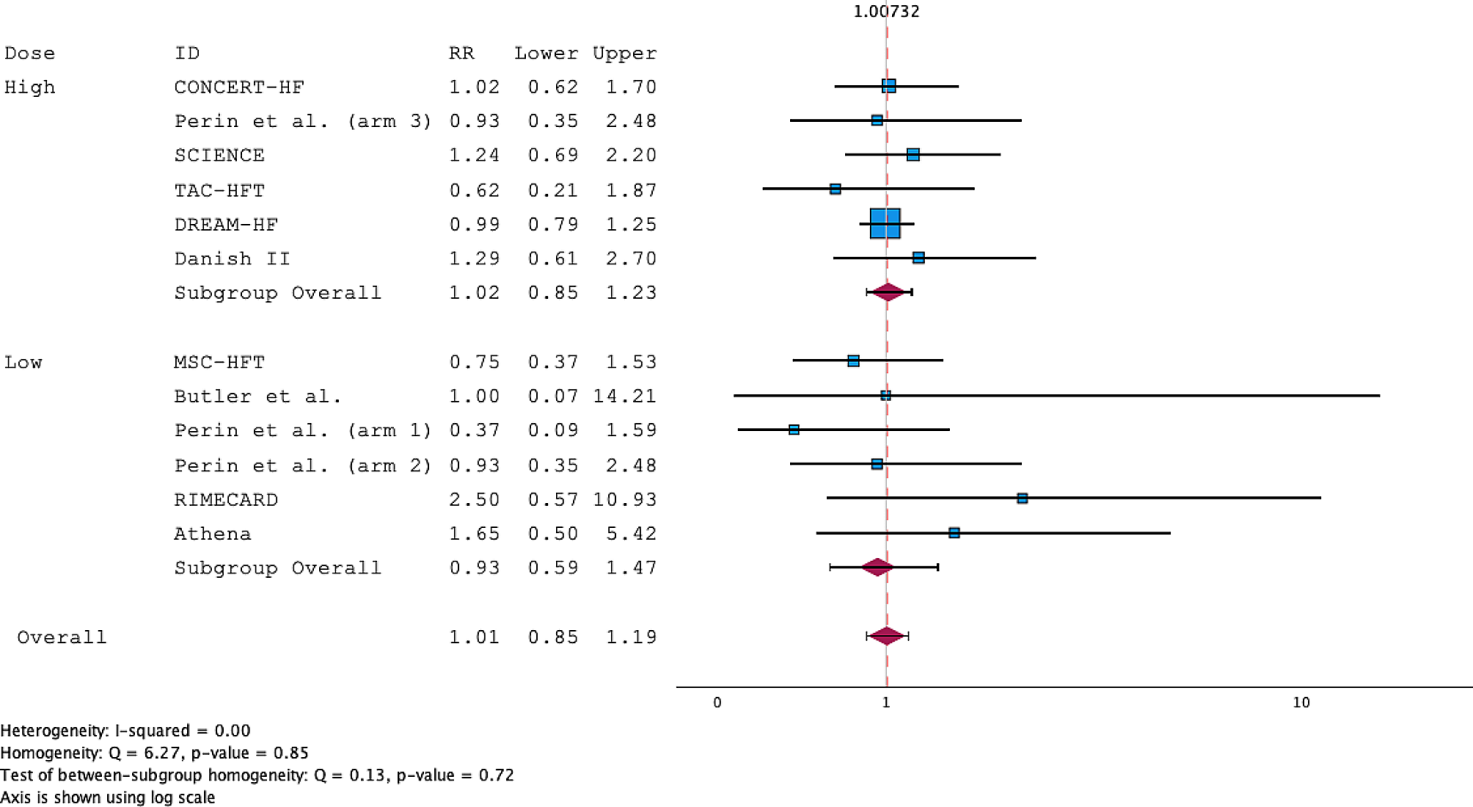
Forest plot of the risk ratio (RR) for MACE in the meta-analysis

**Fig. 4:**
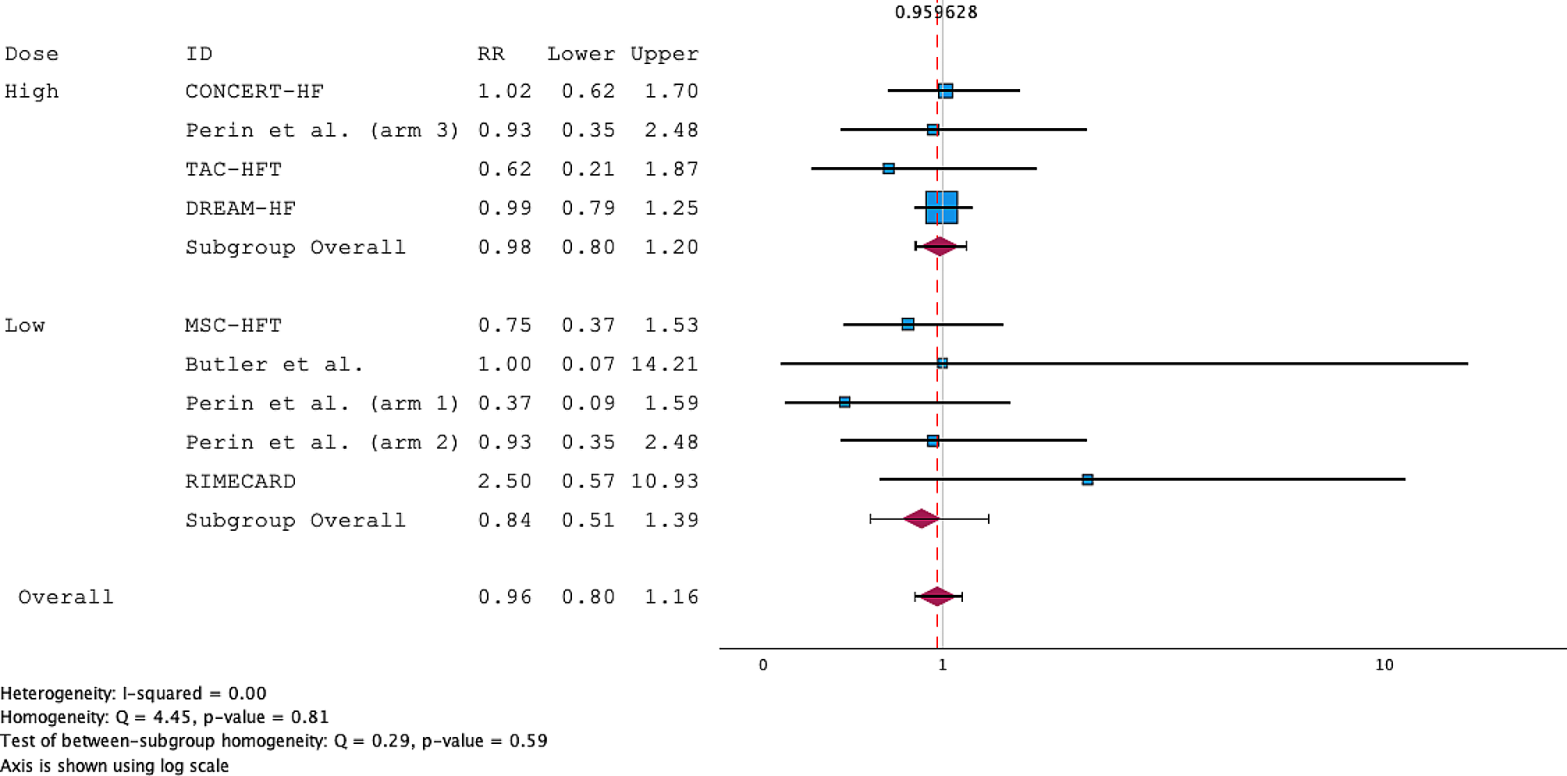
Forest plot of the risk ratio (RR) for MACE meta-analysis excluding ADRC trials

All the RCTs included in the review reported both death and MACE consistently. The overall RRs for death were 0.92 (95% CI; 0.50–1.71) and 0.70 (95% CI; 0.24–2.03) for the low-dose subgroup and 1.06 (95% CI; 0.50–2.26) for the high-dose subgroup (Fig. 2). The studies were homogenous, with an I^2^ = 0.00. A funnel plot and Egger’s regression (*p* = 0.567) showed a low risk of publication bias (Supplementary Fig. 1). For MACE, the pooled RR of MACE after MSC treatment for both high- and low-dose studies was 1.01 (95% CI; 0.85–1.19). Subgroup analysis revealed RRs of 0.93 (95% CI; 0.59–1.47) for the low-dose group and 1.02 (95% CI; 0.85–1.23) for the high-dose group. The studies were highly homogenous, with an I^2^ = 0.00 (Fig. 3). A funnel plot and Egger’s regression (*p* = 0.793) showed a low risk of publication bias (Supplementary Fig. 2). The sensitivity analysis, excluding trials using ADRCs, showed an RR of 0.84 (95% CI; 0.51–1.39) for the low-dose group, which was insignificant (Fig. 4).

#### Left ventricular ejection fraction

**Fig. 5:**
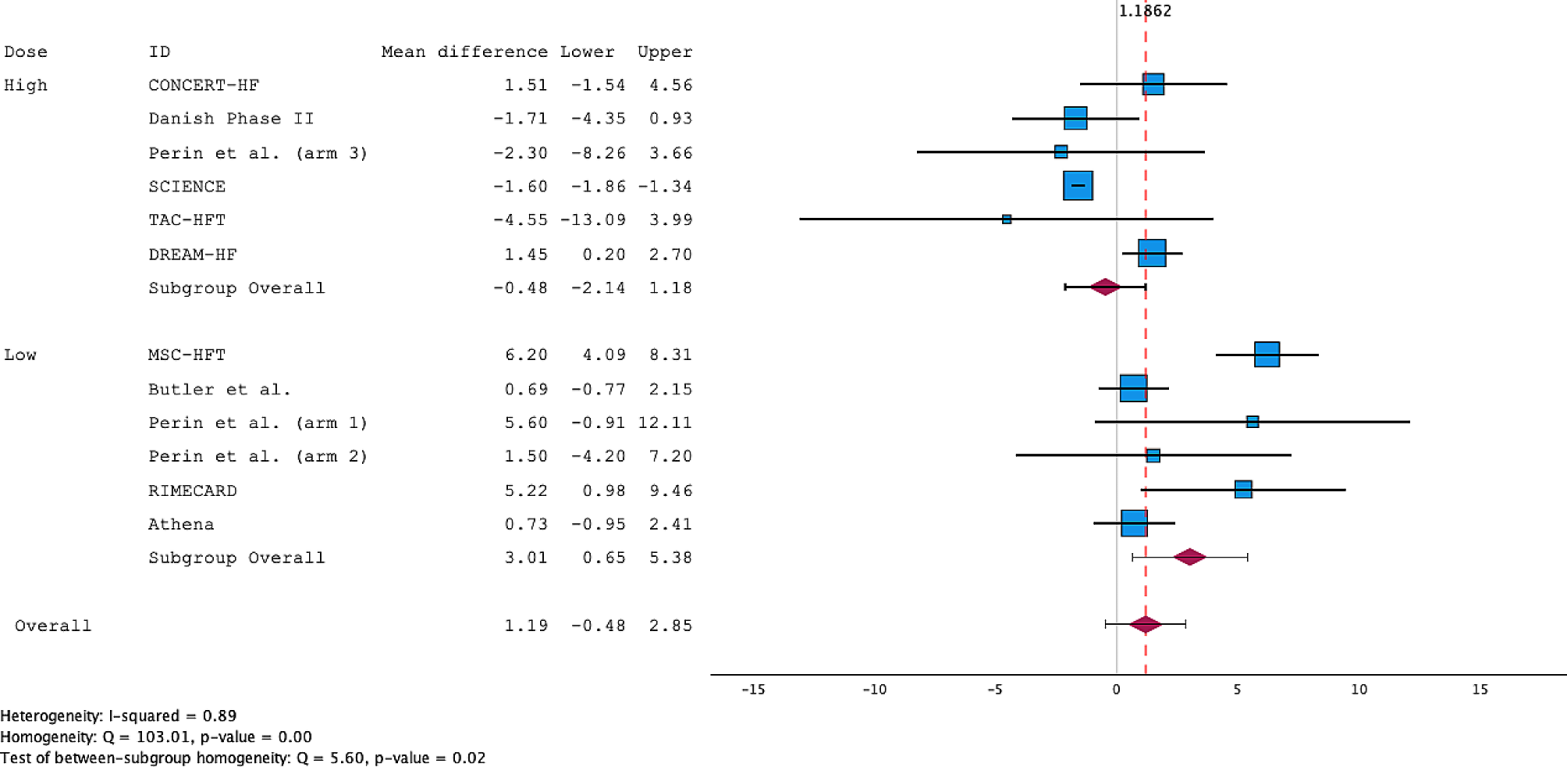
Forest plot of the weighted mean difference (WMD) for LVEF meta-analysis

All the included RCTs reported LVEF and its change from baseline to the follow-up period. Overall, MSC treatment improved the LVEF by 1.19% (95% CI; -0.48-2.85%) compared to the controls, with high heterogeneity (I^2^ = 89). A funnel plot and Egger’s regression showed a low risk of publication bias (*p* = 0.507), with consistent subgroup findings (Supplementary Fig. 3). Subgroup analysis revealed a significant improvement of 3.01% (95% CI; 0.65–5.38%) with low-dose treatment vs. controls and a nonsignificant deterioration of -0.48% (95% CI; -2.14-1.18) with high-dose treatment (Fig. 5).

**Fig. 6:**
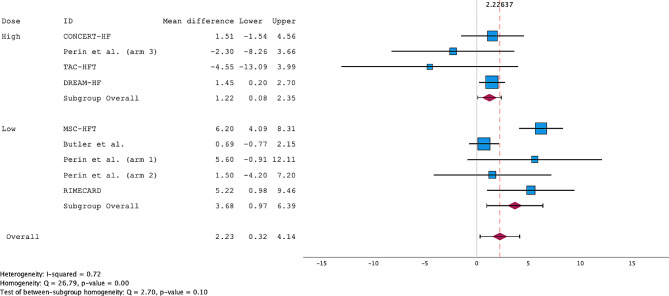
Forest plot of the weighted mean difference (WMD) for LVEF meta-analysis excluding ADRC trials

Interestingly, a sensitivity analysis excluding the ADRC trials showed remarkable improvements in LVEF across both doses (Fig. 6). Further subgroup analysis comparing ADRCs with BMMSCs revealed that ADRCs had a significantly inferior effect (see Supplementary Fig. 4 and Supplementary Fig. 5). Analysis of the LVESV revealed no significant changes across the two dose categories (Supplementary Fig. 6).

#### 6-Minute Walk Distance

**Fig. 7:**
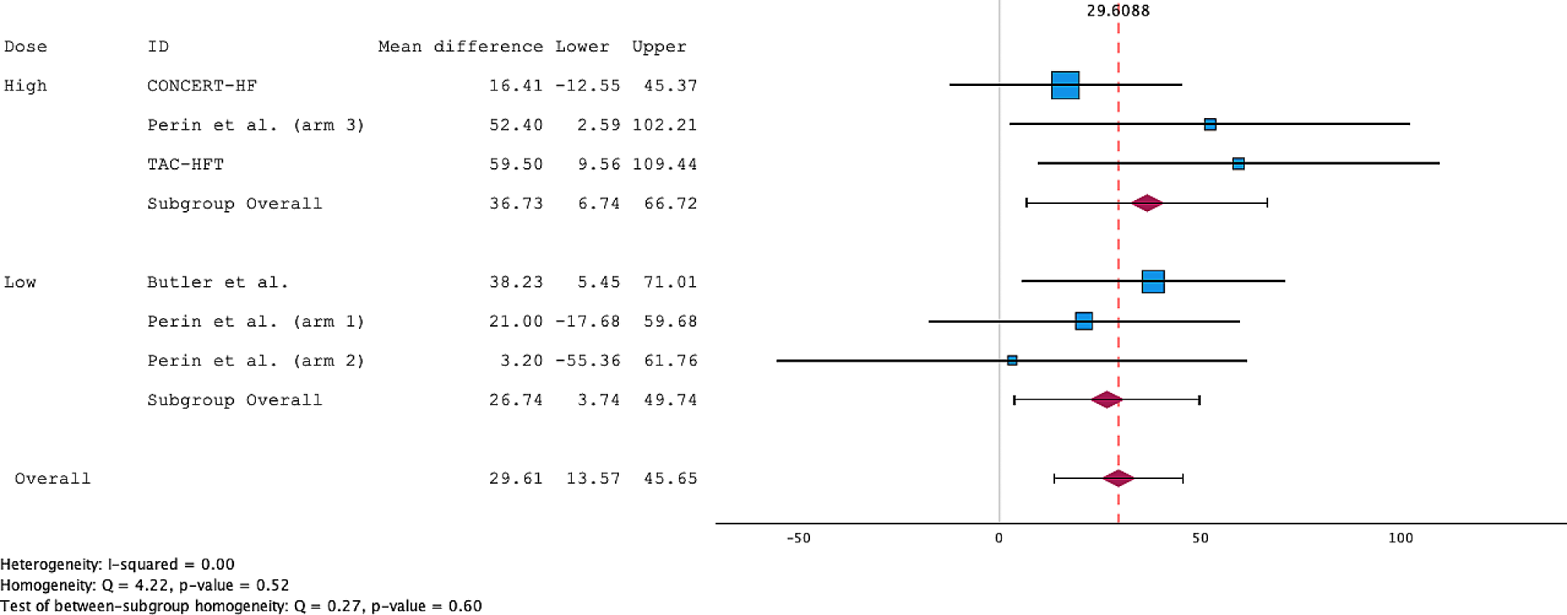
Forest plot of the weighted mean difference (WMD) for the 6MWD meta-analysis

Only four of the included RCTs, with six arms, reported changes from baseline to the follow-up period. The RIMECARD, Athena, and DREAM-HF trials did not use the 6MWD as an endpoint. The remaining trials measured the distance at baseline and follow-up but did not report the change. The corresponding authors were contacted multiple times to provide the necessary data for the analysis and have yet to respond. Overall, MSC treatment increased the 6MWD by 29.61 m (95% CI; 13.57–44.65 m), with low heterogeneity (I^2^ = 0.00) (Fig. 7). A funnel plot and Egger’s regression showed a low risk of publication bias (*p* = 0.789). , with consistent subgroup findings (Supplementary Fig. 7). Subgroup analysis revealed a significant increase in the 6-MWD compared with that of controls (26.74 m; 95% CI; 3.74–49.74 m) with low-dose treatment and 36.73 m (95% CI; 6.74–66.72 m) with high-dose treatment (Fig. 7).

## Discussion

Our systematic review and meta-analysis of eleven RCTs was aimed to establish a dose-response relationship between the safety and efficacy parameters of MSC-based therapy in HFrEF patients. MSCs are rapidly emerging as the cells of choice in regenerative medicine for use as living biodrugs. The main findings of our meta-analysis include the following: (1) All but one of the eleven trials included in the meta-analysis scored high on the Jadad scale, (2) Low-dose MSC treatment was safer and more effective than high-dose MSC treatment in improving efficacy and functional parameters. (3) There was no consensus on defining a low or a high dose in the published RCTs; and (4) BM-derived MSCs were more efficacious than either AD- or UC-derived MSCs.

Different mechanisms contribute to heart failure with preserved ejection fraction (HFpEF) than to HFrEF, and the mechanisms by which cell therapy drives its effects under both conditions seem to differ; therefore, we limited our analysis to the population with HFrEF [39, 40]. We only included MSC injection as the sole treatment to eliminate confounding effects from other interventions. The analysis indicated that low—and high-dose MSC treatments are safe in terms of death and MACE occurrence. Moreover, the results obtained from the sensitivity analysis showed a tendency toward protective effects with low-dose MSC treatment. MSC safety in heart disease and heart failure has been well documented in trials and studies investigating safety in greater depth. Hence, we only analyzed the occurrence of MACE [6, 41–43].

Despite extensive research on MSCs in preclinical and clinical studies, more effort must be made to establish a cell dose-response relationship and determine the optimal cell dose for the desired outcomes. Dose, besides Volume of distribution (Vd) and Clearance, is one of the primary pharmacokinetic parameters that must be defined well for all pharmaceuticals and biopharmaceuticals, including cells as living biodrugs, to achieve optimal pharmacodynamics. Hence, defining the optimal dose will ensure that the clinical use of MSC-based therapy is successful. Their innate heterogeneity and multifarious mechanism of action, which entail several intricate inter- and intracellular pathways, will continue to obscure a clear dose-response relationship for MSCs until well-defined and well-designed dose-escalation studies are performed as a concerted effort between research groups involved therein to ensure that low, moderate and high doses are well-defined. The currently reported contradictory data about the relationship between cell dose and clinical outcomes may be attributed to the inconsistent and diverse classification of low, moderate, and high doses. Given the lack of a consensus on defining cell dose in the published RCTs, our arbitrary classification of low and high cell doses is based on our observations of the doses used during these RCTs in HF patients to facilitate our meta-analysis.

The dose-response efficacy analysis revealed an inverse relationship, as evidenced by improvements in the LVEF and LVESV. However, only five of the included RCTs reported changes in the 6-MWD, which included three arms in the high-dose subgroup and only two in the low-dose subgroup. Consequently, despite being encouraging, the results from the 6-MWD suffer from apparent data deficiency and require cautious interpretation. Preclinical studies support the inverse dosage response, as exceeding particular dosing thresholds can affect cell retention, survival, and function [44, 45]. Our results align with the PROMETHEUS and POSEIDON trials, as both used MSCs in HFrEF. The POSEIDON trial demonstrated that cell therapy had an inverse dose-response relationship with LVEF improvements. It was reported that 20 million cells had a more significant effect than 200 million cells, which could not be attributed to differences in baseline values. Similarly, the PROMETHEUS trial revealed substantial increases in LVEF in the low-dose (20 million cells) group compared to the high-dose (200 million cells) group. Some trials using cells other than MSCs support the inverse dose-response relationship [46, 47].

Some trials also support a direct dose-response relationship [19, 48]. For example, with 20 million and 100 million cells at low and high doses, respectively, the TRIDENT trial reported significant LVEF improvement only in patients treated with 100 million cells, indicating a direct dose-response relationship [48]. As we used 100 million cells as a low-dose treatment in our meta-analysis, there is a possibility that there is an upper limit for the cell dose beyond which the direct dose-response relationship changes into an inverse dose-response relationship. In addition to cell number, cell concentration may also affect the response. Higher cell concentrations may result in higher viscosity, leading to sedimentation, uneven injection flow, and cell death due to limited oxygen diffusion and increased shear forces [49, 50]. It is thus suggested that increasing the dose is beneficial until a “ceiling dose” is reached, beyond which an increase in the cell dose does not add any benefits. These observations necessitate ascertaining this dose-response relationship to guide future large phase III trials.

ADRCs are considered better candidates for therapeutic applications than their counterparts from other tissue sources due to their ease of availability in large quantities. Additionally, adipose tissue is more abundant and accessible to collect with lower invasiveness. However, using one cell population over another may benefit patients with specific pathologies [51–53]. Several clinical and preclinical studies have reported the superiority of ADRCs [54, 55]. However, there is limited cardiomyogenic differentiation [56, 57]. However, akin to BM-MSCs, paracrine effects lead to proangiogenic, antiapoptotic, and immunomodulatory effects [58–61]. Although animal studies using ADRCs were very promising and showed significant improvements in cardiac function [62], phase I studies in patients with heart failure generally showed no significant improvements in cardiac functional parameters. However, they are safe [63, 64]. Our findings indicate that BM-MSCs might be superior to ADRCs in terms of efficacy in HFrEF patients. Nevertheless, these findings should be confirmed with further, more extensive phase II/III studies primarily focusing on cell dose gradation and dose escalation to develop a dose-response relationship for fast-emerging MSCs as living biodrugs.

Despite encouraging safety and efficacy data leading to improved functional outcomes regarding the 6-MWD, the study has limitations. For example, inconsistencies in reporting functional parameters such as the 6-MWD and New York Heart Association (NYHA) class data during the follow-up period made it difficult to assess these parameters accurately. Additionally, the study did not include multiple trials that examined the dose-response effect by following a dose escalation protocol, mainly due to the lack of a control arm. Moreover, heterogeneity in clinical design, such as the route of administration, source, sampling site, and follow-up period, may affect the reliability of and bias the results. However, the study results provide preliminary insight into the dose-response relationship to guide the future design of clinical trials.

## Conclusions

In conclusion, our results support low-dose MSC treatment in HFrEF patients for improved safety and efficacy. Including phase II/III RCTs involving HFrEF patients treated with MSC-based therapy reduced the risk of bias. Our data provide insight into determining the appropriate dosage before proceeding with large phase III trials. Subgroup analysis suggested the superiority of BM-derived MSCs over ADSCs. Nevertheless, these data warrant further investigation to establish a dose-response relationship using well-defined categorization of low, medium, and high doses of cells.

### Electronic supplementary material

Below is the link to the electronic supplementary material.

Supplementary Material 1

## Data Availability

The datasets used and analyzed during the current study are available from the corresponding author upon reasonable request.

## Abbreviations

CMs: Cardiomyocytes
HF: Heart failure
HFrEF: HF patients with reduced ejection fraction
LVEF: Left ventricular ejection fraction
LVESV: Left ventricular end-systolic volume
MACE: Major adverse cardiac events
MSCs: Mesenchymal stem cells
6-MWD: 6-Minute walk distance
RCTs: Randomized controlled trials
