# Supplementary material for: Dose-response relationship of MSCs as living Bio-drugs in HFrEF patients: a systematic review and meta-analysis of RCTs
Source: Stem Cell Res Ther. 2024 Jun 13;15:165. doi: 10.1186/s13287-024-03713-4 (PMC11170815; doi:10.1186/s13287-024-03713-4)
Supplement: Supplementary file 1 — Supplementary Material 1 [file 13287_2024_3713_MOESM1_ESM.docx]

**Supplementary material**

**Appendix 1. Supplementary figures.**

**Supplementary Figure 1.** Funnel plot and Egger’s regression for death meta-analysis.


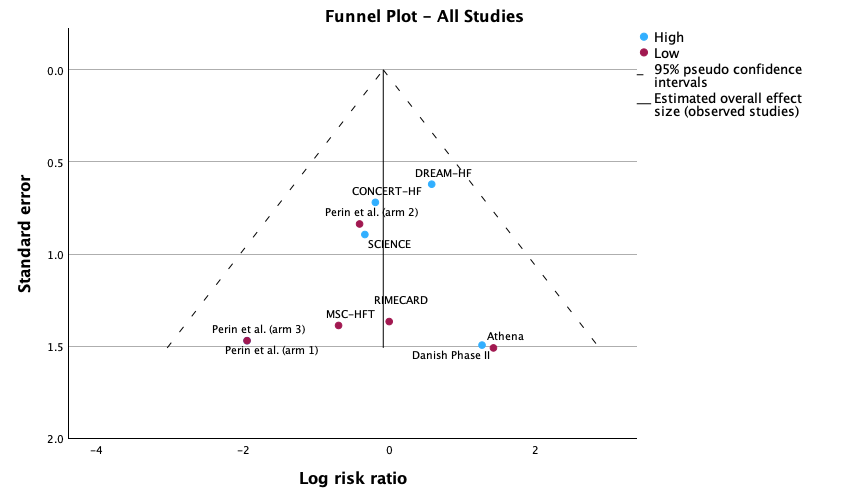


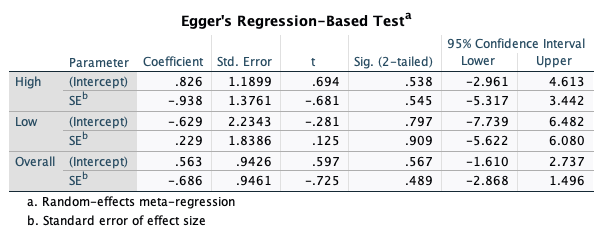


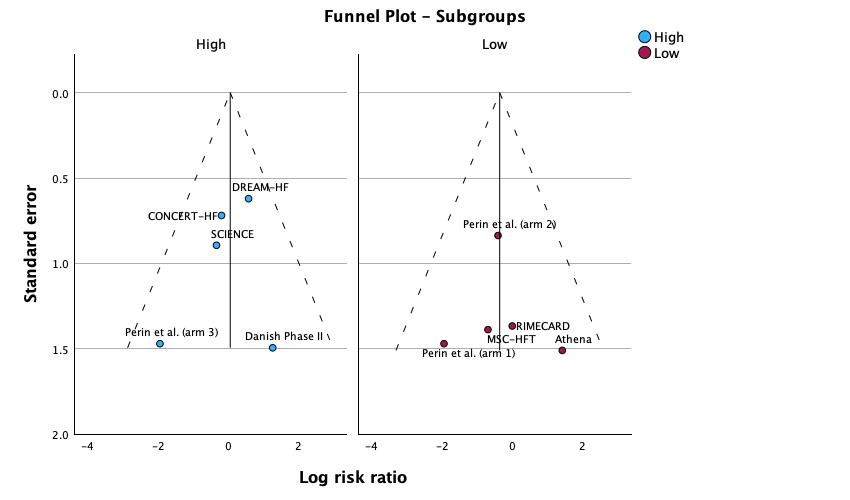


**Supplementary Figure 2:** Funnel plot and Egger’s regression for MACE meta-analysis.


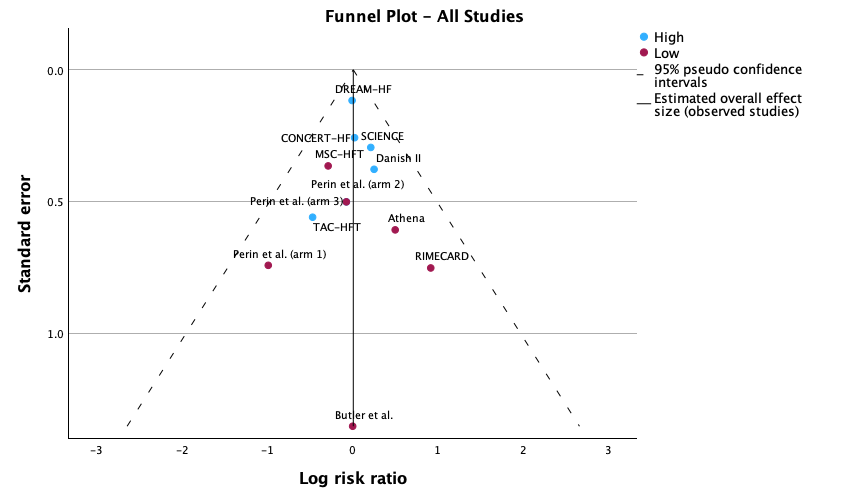


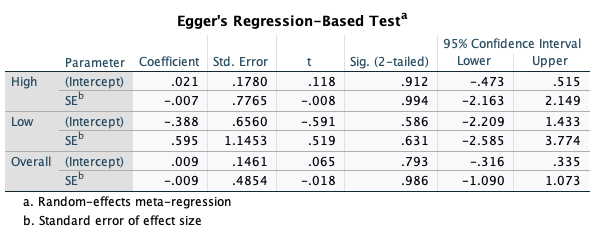


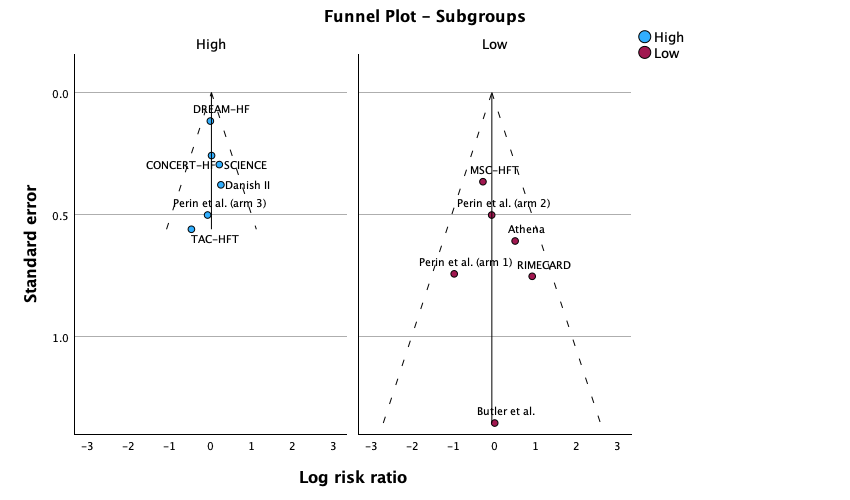


**Supplementary Figure 3.** Egger’s regression test with subgroup funnel plots of LVEF.


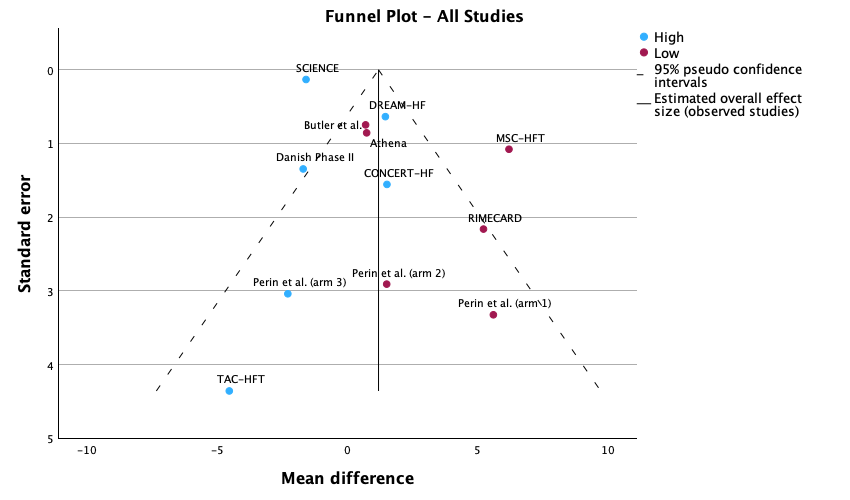


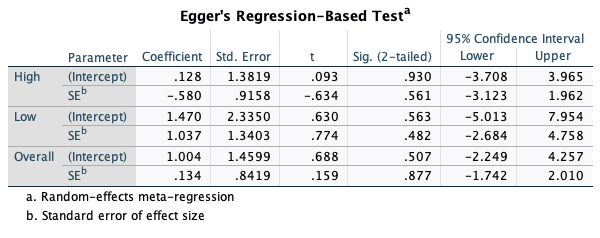


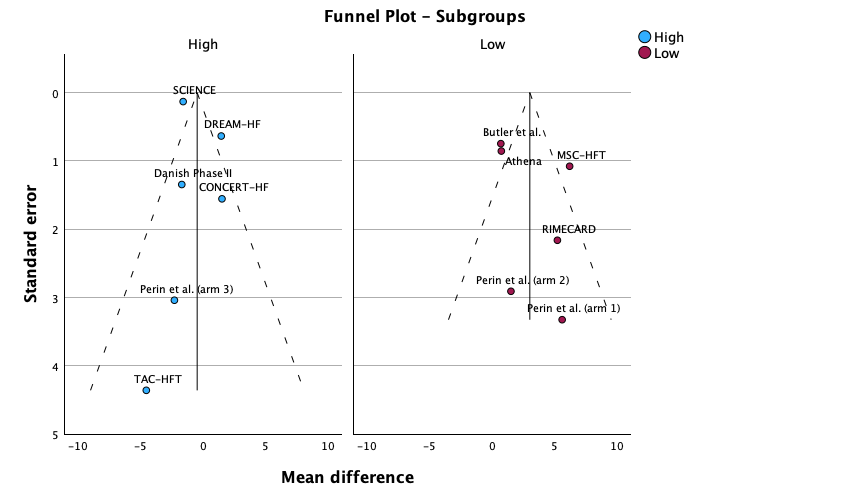


**Supplementary Figure 4.** Subgroup analysis of high-dose studies comparing improvement in LVEF between ADRCs and BMMSCs.


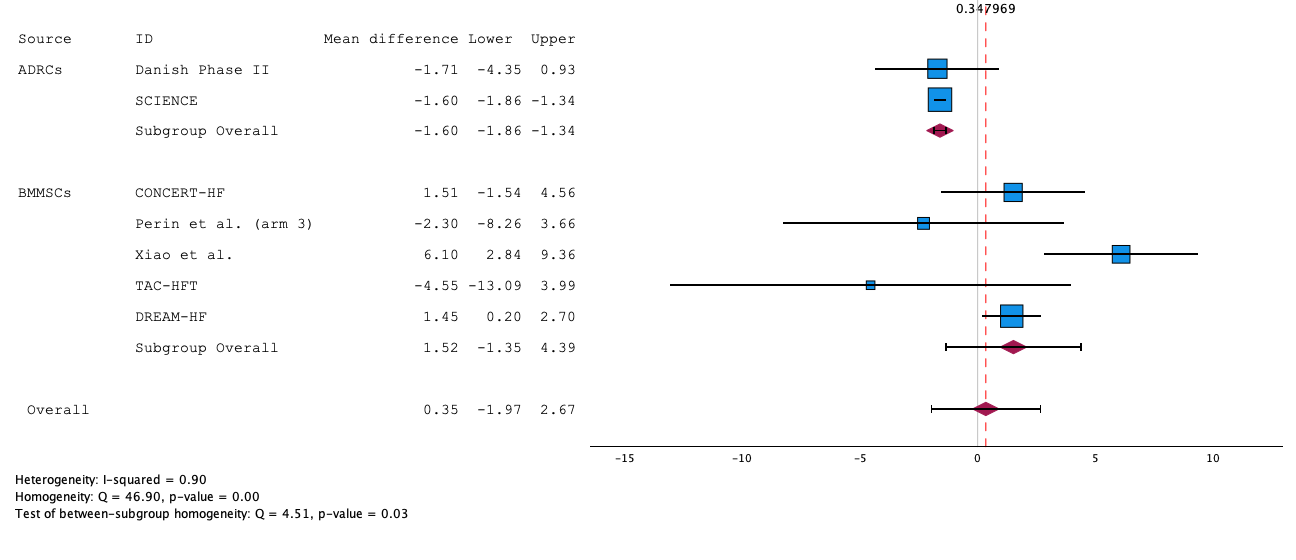


**Supplementary Figure 5.** Subgroup analysis of low-dose studies comparing improvement in LVEF between ADRCs and BMMSCs.


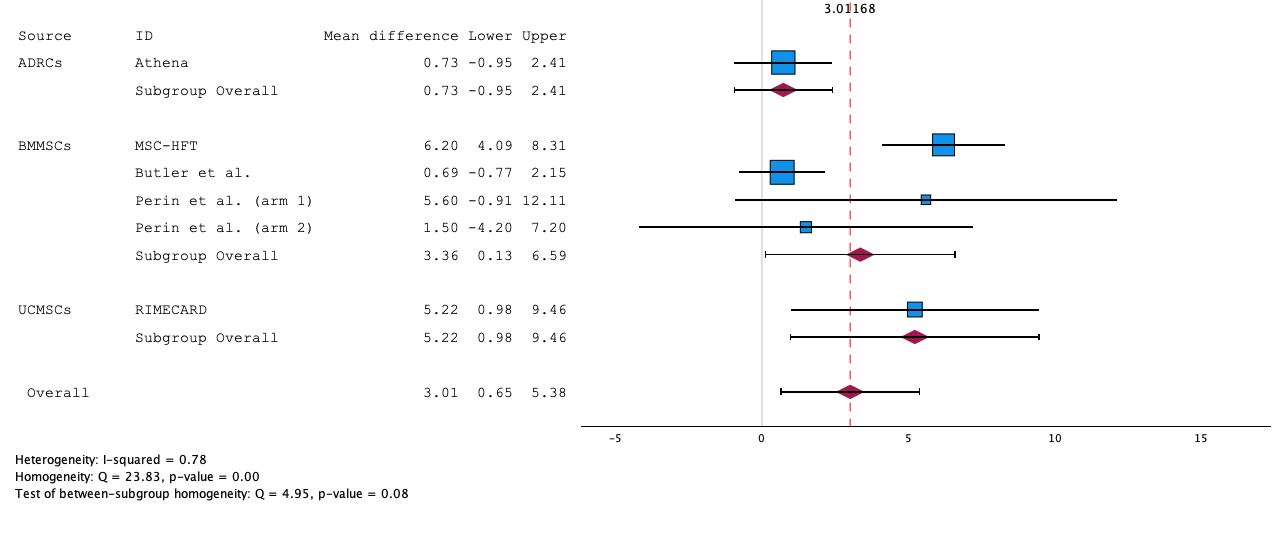


**Supplementary Figure 6.** Forest plot of the weighted mean difference (WMD) for the LVESV


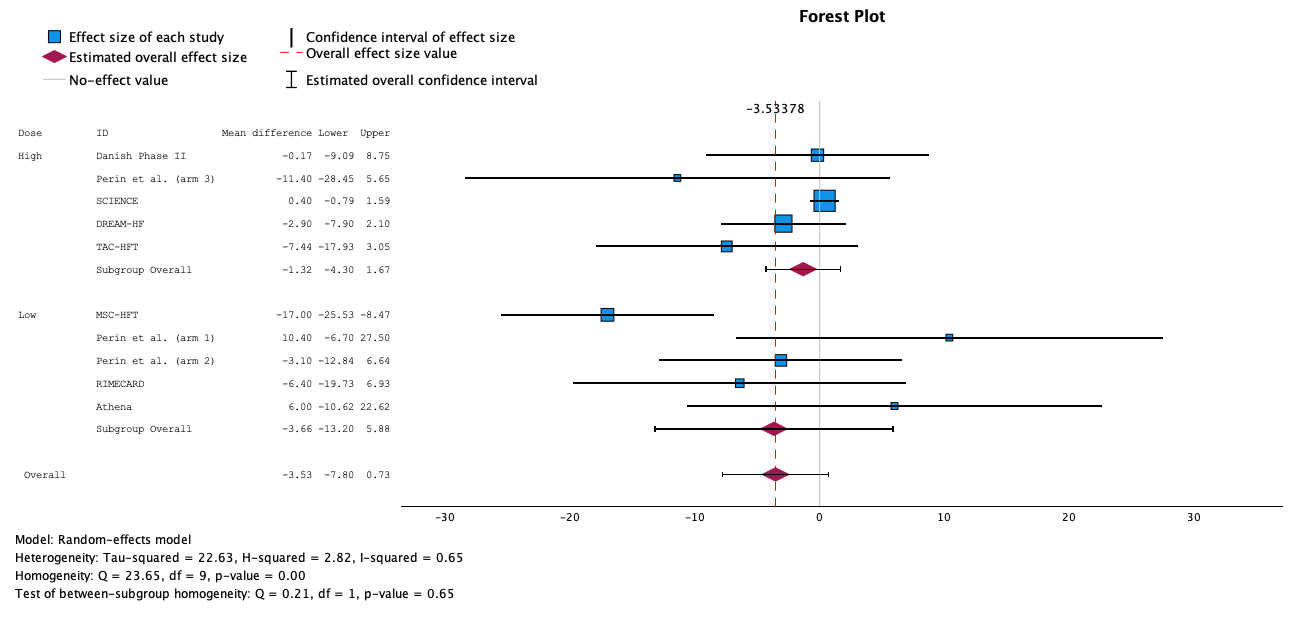


**Supplementary Figure 7.** Funnel plot and Egger’s regression test with subgroup funnel plots for the 6-MWD.


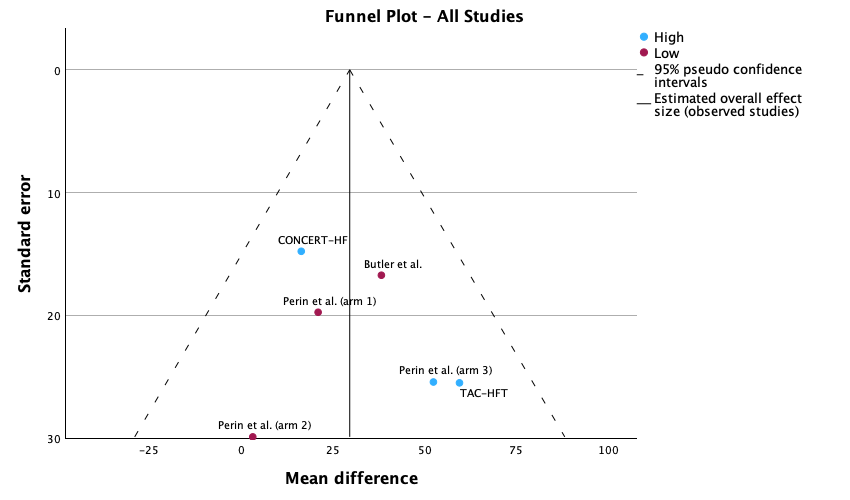


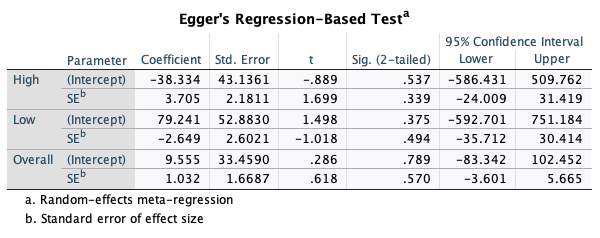


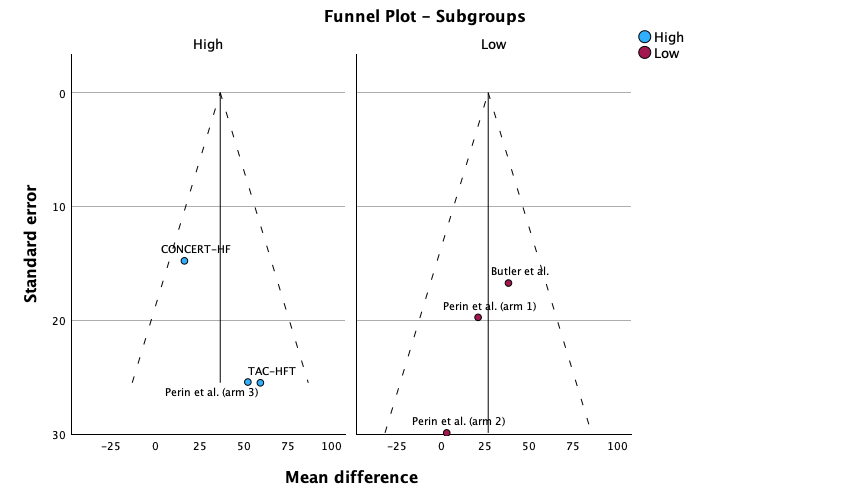


**Appendix 2.** Definition of Major Adverse Cardiac Events (MACE).

We defined MACE as the occurrence of any of the following:

- death or,
- hospitalization due to adverse events, including:
  - worsening heart failure,
  - ischemic heart disease,
  - cardiac arrest,
  - arrhythmias (i.e., A-Fib, A-Flut, V-Fib, V-Tach, 3rd-degree block, and LBBB),
  - cardiogenic shock,
  - cardiac tamponade,
  - revascularization procedures,
  - and ICD/CRT implantation/revision

**Appendix 3.** Analysis protocol.

This meta-analysis strictly followed the guidelines of the Cochrane Handbook for Systematic Reviews of Interventions.

The analysis was conducted using the SPSS version 28 statistical package (SPSS Inc., Chicago, IL, USA).

The protocol for the analysis will be as follows:

- The data needed for the analysis were extracted and plotted (Data Table 1). All the available data from the papers and their supplementary material were extracted.
- The corresponding authors of papers that did not provide the needed data in the original article or the supplementary material were contacted to provide the data. Reminders were sent if the authors forgot to respond.
- If the corresponding authors did not respond, data feasible for imputation were imputed following the Cochrane Handbook for Systematic Reviews of Interventions guidelines.
- If data imputing values were unavailable, the study was excluded from the analysis.
- If the authors presented the data in figure form instead of in text or tables, the corresponding authors were first contacted. In the case of no response, the webplotdigitizer (<https://automeris.io/WebPlotDigitizer/>) was used to extract the data only if the figure offered the final data needed in complete form (i.e., mean difference standard deviation). If the data were in a form that required further imputation, the study was excluded from the respective analysis.
- An unstandardized mean difference meta-analysis using a restricted maximum likelihood inference to treat protocol.

**Left ventricular ejection fraction (LVEF) analysis:**

- The MSC-HFT, Perin et al., Athena, RIMECARD, TAC-HFT, CONCERT-HF, SCIENCE, and DREAM-HF trials provided all the data needed in the form of the mean difference standard deviation.
- Butler et al. provided the mean difference without its standard deviation. The corresponding authors were contacted but received no response. The standard deviation was calculated as described in the Cochrane Handbook for Systematic Reviews of Interventions, section 6.5.2.3 (<https://training.cochrane.org/handbook/current/chapter-06#section-6-5-2-3>).
- The Danish phase II trial reported the mean difference and standard deviation in a figure form. The corresponding authors were contacted but received no response. The webplotdigitizer was used to extract the data.
- Xiao et al. provided the mean difference without its standard deviation. The corresponding authors were contacted but received no response. The standard deviation was calculated as described in the Cochrane Handbook for Systematic Reviews of Interventions, section 6.5.2.8 (<https://training.cochrane.org/handbook/current/chapter-06#section-6-5-2-8>). However, the study was not included due to its low quality.

**6-minute walking distance (6-MWD):**

- Only the CONCERT-HF, Perin et al., TAC-HFT, and Butler et al. trials reported the mean difference in the 6-MWD. Of those, only the CONCERT-HF and TAC-HFT trials provided the standard deviation with the mean difference.
- Butler et al. and Perin et al. provided the mean difference without its standard deviation. The corresponding authors were contacted but received no response. The standard deviation was calculated as described in the Cochrane Handbook for Systematic Reviews of Interventions, section 6.5.2.3 (<https://training.cochrane.org/handbook/current/chapter-06#section-6-5-2-3>).

**Left ventricular end colic volume (LVESV):**

- The MSC-HFT, Perin et al., Athena, RIMECARD, SCIENCE, Danish Phase II, and DREAM-HF trials provided LVEV measurements.
- The mean difference with its standard deviation was reported for only the MSC-HFT, Perin et al., Athena SCIENCE, and Danish phase II trials.
- RIMECARD and DREAM-HF provided the mean difference without its standard deviation. The corresponding authors were contacted but received no response. The standard deviation was calculated as described in the Cochrane Handbook for Systematic Reviews of Interventions, section 6.5.2.3 (<https://training.cochrane.org/handbook/current/chapter-06#section-6-5-2-3>).
